# Regulation of NADPH Oxidase-Mediated Superoxide Production by Acetylation and Deacetylation

**DOI:** 10.3389/fphys.2021.693702

**Published:** 2021-08-12

**Authors:** Ning Xia, Stefan Tenzer, Oleg Lunov, Martin Karl, Thomas Simmet, Andreas Daiber, Thomas Münzel, Gisela Reifenberg, Ulrich Förstermann, Huige Li

**Affiliations:** ^1^Department of Pharmacology, Johannes Gutenberg University Medical Center, Mainz, Germany; ^2^Department of Immunology, Johannes Gutenberg University Medical Center, Mainz, Germany; ^3^Institute of Pharmacology of Natural Products and Clinical Pharmacology, University of Ulm, Ulm, Germany; ^4^Department of Optical and Biophysical Systems, Institute of Physics ASCR, Prague, Czechia; ^5^Department of Cardiology, Cardiology I, Johannes Gutenberg University Medical Center, Mainz, Germany

**Keywords:** NADPH oxidase, Rac1, sirtuin 1, acetylation, resveratrol, histone deacetylase, histone acetyltransferase

## Abstract

Oral treatment of apolipoprotein E-knockout (ApoE-KO) mice with the putative sirtuin 1 (SIRT1) activator resveratrol led to a reduction of nicotinamide adenine dinucleotide phosphate (NADPH) oxidase activity in the heart. In contrast, the SIRT1 inhibitor EX527 enhanced the superoxide production in isolated human polymorphonuclear granulocytes. In human monocytic THP-1 cells, phorbol ester-stimulated superoxide production was enhanced by inhibitors of histone deacetylases (HDACs; including quisinostat, trichostatin A (TSA), PCI34051, and tubastatin A) and decreased by inhibitors of histone acetyltransferases [such as garcinol, curcumin, and histone acetyltransferase (HAT) Inhibitor II]. These results indicate that protein acetylation and deacetylation may represent crucial mechanisms regulating NADPH oxidase-mediated superoxide production. In cell-free systems, incubation of recombinant Rac1 with SIRT1 resulted in decreased Rac1 acetylation. Mass spectrometry analyses identified lysine 166 (K166) in Rac1 as a residue targeted by SIRT1. Deacetylation of Rac1 by SIRT1 markedly reduced the interaction of Rac1 with p67phox in *in vitro* assays. Computational modeling analyses revealed that K166 deacetylation of Rac1 led to a 5-fold reduction in its binding affinity to guanosine-5'-triphosphate, and a 21-fold decrease in its binding potential to p67phox. The latter is crucial for Rac1-mediated recruitment of p67phox to the membrane and for p67phox activation. In conclusion, both SIRT1 and non-sirtuin deacetylases play a role in regulating NADPH oxidase activity. Rac1 can be directly deacetylated by SIRT1 in a cell-free system, leading to an inhibition of Rac1-p67phox interaction. The downstream targets of non-sirtuin deacetylases are still unknown. The *in vivo* significance of these findings needs to be investigated in future studies.

## Introduction

Oxidative stress, defined as an excessive production of oxidants over antioxidants, results in an oxidizing redox state in the body (Drummond et al., 2011). Oxidative stress is closely related to all cardiovascular risk factors, such as, hypertension, hyperlipidemia, and diabetes. A number of researches indicate that oxidative stress is involved in the formation of atherosclerotic plaques. Thus, oxidative stress may represent an attractive target for cardiovascular disease prevention or therapy (Li et al., 2013, 2014).

Among all the enzyme systems that produce reactive oxygen species (ROS), NADPH oxidases are likely to be the major ROS sources in the cardiovascular system (Bedard and Krause, 2007; Drummond et al., 2011; Lassegue et al., 2012). Nox, which is the catalytic subunit of NADPH oxidases, constitutes the Nox family with seven homologs, Nox1 to Nox5, Duox1 and Duox2. The phagocytic NADPH oxidase enzyme complex is comprised of two transmembrane subunits (Nox and the p22phox) and several regulatory cytosolic subunits, including p47phox, p67phox, p40phox, and a small GTPase Rac (Rac1 or 2). The NADPH oxidase isoforms are defined by the nature of their core catalytic subunits (Nox1–5) as well as their suite of regulatory subunits (Drummond et al., 2011). Nox4 and Nox5, for instance, do not require the cytosolic subunits for activity. The classic NADPH oxidase, Nox2 (gp91phox), was originally found in phagocytes. Nevertheless, Nox2 is expressed also in many other cell types, including endothelial cells, adventitial fibroblasts, cardiomyocytes, as well as monocytes and T cells infiltrated in cardiovascular tissues (Bedard and Krause, 2007; Drummond et al., 2011). Cytochrome b558 is formed by Nox2 and p22phox. Upon stimulation, the cytosolic subunit translocate to the plasma membrane, leading to activation of the NADPH oxidase enzyme complex (Brandes and Kreuzer, 2005).

Typically, the translocation of the cytosolic subunits is initiated by phosphorylation of p47phox. Tumor necrosis factor-α (TNF-α) and protein kinase C (PKC)-activating phorbol esters activate endothelial NADPH oxidase in a p47phox-dependent manner. Angiotensin II stimulation leads to biphasic activation of NADPH oxidases. Whereas the initial activation is a consequence of PKC-mediated p47phox phosphorylation, the later phase is the result of a signaling cascade involving EGF receptor transactivation, PI3K, and subsequent Rac activation (Brandes and Kreuzer, 2005).

Resveratrol (3,5,4′-trihydroxy-trans-stilbene) is a natural polyphenolic phytoalexin produced by a variety of plant species (Li et al., 2012; Xia et al., 2014, 2016). As the major polyphenol presented in red wine, resveratrol has been correlated with lower incidence of myocardial infarction in France compared to other countries, which is the so-called “French paradox.” In our previous study, resveratrol was shown to decrease oxidative stress in atherosclerosis-prone apolipoprotein E-knockout (ApoE-KO) mice by limiting ROS production and accelerating ROS detoxification (Xia et al., 2010). In addition, treating with resveratrol decreased the expression of Nox2 and Nox4 in the heart of ApoE-KO mice (Xia et al., 2010). In the present study, we provide evidence that resveratrol also inhibits the activity of NADPH oxidase. Because resveratrol can increase the expression and activity of the histone/protein deacetylase sirtuin 1 (SIRT1), we hypothesized that the resveratrol-induced inhibition of NADPH oxidase activity may involve SIRT1-mediated deacetylation of the NADPH oxidase regulatory subunits. We started the study by using ApoE-KO mice, because these animals are known to have higher NADPH oxidase activity than wild-type mice (Hsich et al., 2000).

## Materials and Methods

### Animals and Treatment

Six-month old male ApoE-KO mice (Charles River Laboratories, Sulzfeld, Germany) were treated with resveratrol (trans-3,4′, -5-trihydroxystilebene) at doses of 30 or 100 mg/kg through oral gavage for 7 days as previously described (Xia et al., 2010). Resveratrol was obtained from Cayman Chemical (Ann Arbor, Michigan, United States). The animal experiment was approved by the responsible regulatory authority (Landesuntersuchungsamt Rheinland-Pfalz; G07-1-032) and was performed in accordance with the German animal protection law and the guidelines for the use of experimental animals as stipulated by the Guide for the Care and Use of Laboratory Animals of the National Institutes of Health.

### NADPH Oxidase Activity Assay With Lucigenin Chemiluminescence

Lucigenin chemiluminescence assay was used to measure membrane NADPH oxidase activity. Although lucigenin is known to undergo redox cycling at higher concentrations, lucigenin at 5 μM was validated for detecting superoxide production (Li et al., 1998; Skatchkov et al., 1999; Dikalov et al., 2007). Heart was homogenized (glass/glass) in a homogenization buffer containing 50 mM Tris•HCl pH 7.4, 2 mM dithiothreitol, and a protease inhibitor cocktail (Roche) and centrifuged at 2,000 *g* for 5 min at room temperature. The supernatant was removed, and the remaining lysate was centrifuged at 20,000 *g* for 20 min at 4°C. After the second centrifugation, the supernatant was removed and centrifuged at 100,000 *g* for 60 min at 4°C. Finally, the pellet was resuspended in homogenization buffer (without dithiothreitol) and further diluted in PBS to a final protein concentration of 0.2 mg/ml for the assay. Five micrometer lucigenin-derived chemiluminescence of the membrane suspensions was detected by a Lumat LB 9507 (Berthold) in the presence of 200 μM NADPH.

### *In vitro* Acetylation and Deacetylation of Rac1

For *in vitro* acetylation, 1 μg GST-tagged recombinant Rac1 (Cytoskeleton Inc., Denver, United States) was incubated with acetyl-CoA (20 μM, Active Motif) and p300 acetyltransferase (100 ng, Active Motif) at 30°C for 30 min. The sample was then incubated with deacetylation buffer (25 mM Tris•HCl, pH 8.0, 137 mM NaCl, 2.7 mM KCl, and 1 mM MgCl_2_/1 mg/ml BSA), 1 mM NAD^+^, and the active recombinant SIRT1 (100 units; Biozol, MBL Inc., Woburn) at 37°C for 1 h to initiate the deacetylation reaction. The reaction mixture was used for subsequent immunoblotting, pull-down or mass spectrometry analyses. In case of immunoblotting, the reaction mixtures were subjected to SDS-PAGE and immunoblotted with antibodies against SIRT1 (Santa Cruz), Rac1 (Millipore), p67phox (Epitomics), or acetylated lysine (Cell Signaling).

### Mass Spectrometry

Rac1 (de)acetylation reaction mixtures (see above) were subjected to SDS-PAGE, and the gel was stained with Coomassie blue R-250 (Sigma). Relevant protein bands were excised and sliced into small pieces followed by in-gel digestion with trypsin (for details see Supplementary data). Digested peptides were transferred into autosampler vials for LC-MS analysis using a Waters Q-TOF Premier API system (Tenzer et al., 2011). The LC-MS data were processed using PROTEINLYNX GLOBAL SERVER, Ver. 2.4. (Waters), and protein identifications were assigned by searching a custom compiled database containing human proteins (Uniprot Swissprot Release 2011-08, 20,256 entries) supplemented with known possible contaminants (trypsin, GST). All identified peptide sequences were verified by manual interpretation of the fragment spectra.

### *In vitro* Rac1-p67phox Interaction and Rac1 Pull-Down Experiment

The Rac1 deacetylation reaction mixtures (Rac1 ± SIRT1) were incubated with 3 μg recombinant p67phox (OriGene) at 4°C for 1 h. Then, Rac1 pull-down was performed with 40 μl 50% glutathione-agarose beads (BD Biosciences), as Rac1 was the only protein carrying a GST-tag in the reaction mixture. After washing, the pull-down complexes were subjected to SDS-PAGE and immunoblotted with anti-Rac1 (Millipore), anti-p67phox (BD Biosciences), and anti-acetylated lysine (Cell Signaling).

### Computational Modeling

Computational docking and scoring studies of the interaction of Rac1 and acK166Rac1with p67phox were performed using Hex 6.3. Modeling of GTP binding to Rac1 and acK166Rac1 was done using Molegro Virtual Docker 5. The original parameters of blind docking were used in combination with an evaluation algorithm based on binding free energy (ΔG). Structure of the tetratricopeptide repeat (TPR) domain of p67phox in complex with Rac.GTP (Lapouge et al., 2000) was obtained from the Protein Data Bank (1E96; http://www.rcsb.org/pdb/explore.do?structureId=1e96). A molecular model of acK166Rac1 was generated by template LOMETS homology modeling with structure fragmentation and reassembling by replica-exchange Monte Carlo simulations using the I-TASSER standalone package (version 1.1). Three-dimensional models of Rac1-p67phox and acK166Rac1-p67phox complexes with surface charge distribution were created with Molegro Virtual Docker 5.

### Data Analysis

Student’s *t*-test was used for comparison of two groups. One-way ANOVA was used to compare mean values between three or more groups. Values of *p* < 0.05 were considered significantly different.

More methodological details are presented in Supplementary data.

## Results

### Resveratrol Inhibits NADPH Oxidase Activity in ApoE-KO Mouse Heart

**Figure 1:**
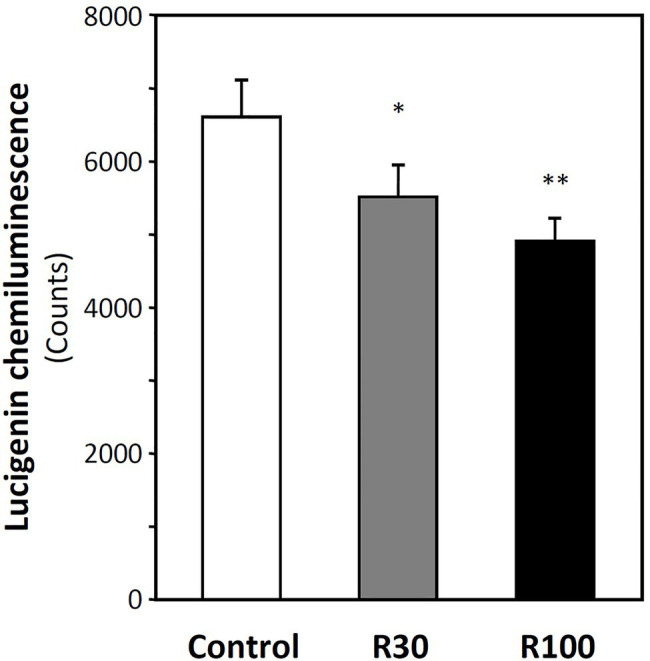
Resveratrol inhibits NADPH oxidase activity. Male apolipoprotein E-knockout (ApoE-KO) mice were treated orally with 30 or 100 mg/kg resveratrol (R30 and R100) for 7 days. Membrane fractions were isolated from the heart. Membrane NADPH oxidase activity was measured by lucigenin (5 μM)-derived chemiluminescence in the presence of 200 μM NADPH. Columns represent mean ± SEM. ^*^p < 0.05 and ^**^p < 0.01 compared with control; *n* = 6 animals in each group.

Apolipoprotein E-knockout mice were treated with resveratrol for 7 days *via* gavage as previously described (Xia et al., 2010). Membrane fractions were isolated from heart lysate, and superoxide production was measured after adding 5 μM lucigenin as readout of NADPH oxidase activity. As shown in Figure 1, resveratrol treatment reduced the levels of ROS. These results are consistent with our previous study in which dihydroethidium was used as a probe and the superoxide-specific oxidation product, 2-hydroxyethidium, was quantified with high-performance liquid chromatography (Xia et al., 2010).

### SIRT1 Inhibition Increase ROS Production in Human PMN

**Figure 2:**
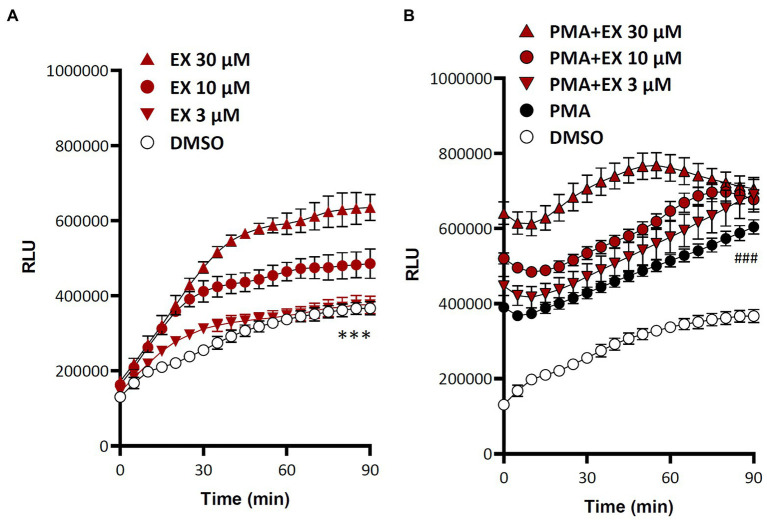
Sirtuin 1 (SIRT1) inhibition increases reactive oxygen species (ROS) production in polymorphonuclear granulocytes (PMN). Human PMNs were isolated from buffy coat and treated with the SIRT1 inhibitor EX527, in the absence **(A)** or presence **(B)** of the phorbol 12-myristate 13-acetate (PMA, 10 nM). ROS production was measured with L-012. Symbols represent mean ± SD, *n* = 6. ^***^p < 0.001, DMSO compared with any other curve **(A)**; ^###^*p* < 0.001, PMA compared with any other curve **(B)**, one-way ANOVA followed by Dunnett’s multiple comparisons test.

Treatment of isolated human polymorphonuclear granulocytes with the SIRT1 inhibitor EX527 (Gertz et al., 2013) led to a concentration-dependent increase in ROS production (Figure 2). Interestingly, this increase could be observed both under basal condition and in cells stimulated with the phorbol ester phorbol 12-myristate 13-acetate (PMA; Figure 2).

### HDAC Inhibition Increases ROS Production in Human THP-1 Cells

**Figure 3:**
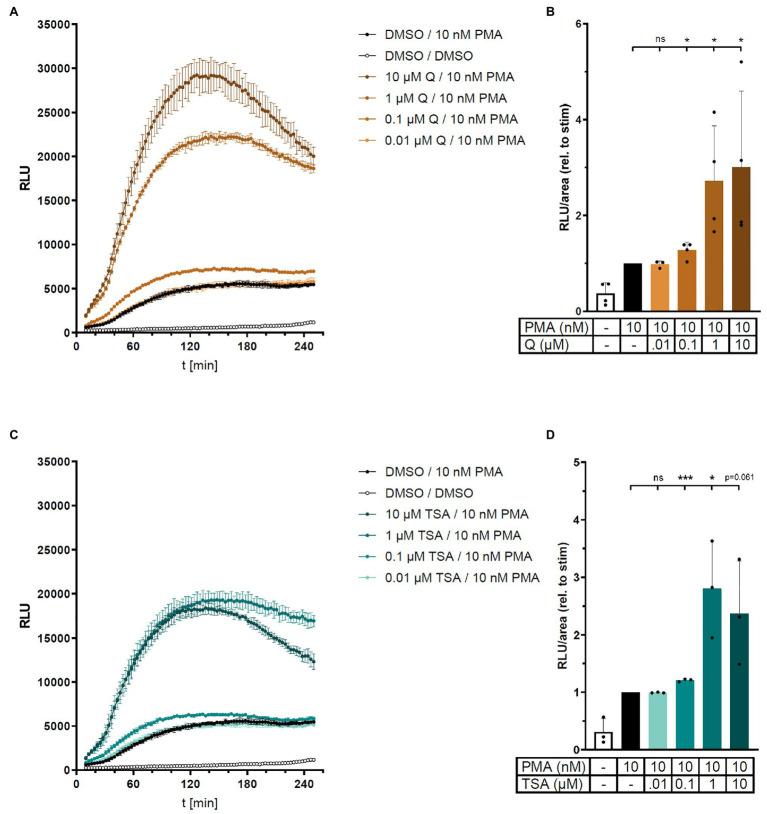
Histone deacetylase (HDAC) inhibition increases ROS production in human THP-1 cells. ROS production in PMA-stimulated human monocytic THP-1 cells was measured in the absence or presence of HDAC inhibitors quisinostat (Q) or trichostatin A (TSA) in an L-012 assay. Relative light units (RLU) were determined over time (**A** and **C**, representative experiments, mean ± SD, *n* = 3 each). **(B)** and **(D)** show combined results from 3 to 4 independent experiments by calculating area under the curve in each experiment (relative to PMA alone). Columns represent mean ± SD. ^*^p < 0.05, ^***^p < 0.001, compared with PMA, unpaired, two-tailed *t*-test.

In the light of the reduced superoxide production by resveratrol (Figure 1) and enhanced ROS production by SIRT1 inhibition (Figure 2), we hypothesized that acetylation and deacetylation could represent general mechanisms regulating NADPH oxidase activity. Therefore, we expanded our study to include also non-sirtuin deacetylases and performed additional experiments in the human monocytic cell line THP-1. In line with the effect of SIRT1 inhibition, broad-spectrum histone deacetylase (HDAC) inhibitors quisinostat and trichostatin A (TSA) increased ROS production in PMA-stimulated but not unstimulated THP-1 cells as measured in an L-012 assay (Figure 3). Importantly, application of either PEG-superoxide dismutase (SOD) or inhibitors of NADPH oxidase was sufficient to quench the luminescence signal in the L-012 assay to basal levels of unstimulated cells, demonstrating that ROS measured in this assay indeed originates from NADPH oxidase activity (Supplementary Figures S1, S2). HDAC inhibition potentiates ROS production by NADPH oxidase specifically in the presence of stimulation (PMA), potentially linking deacetylation to NADPH oxidase priming.

To identify the HDAC family members involved in the regulation of NAPDH activity, isoform-specific HDAC inhibitors were applied in the L-012 assay. Interestingly, PCI34051 (an inhibitor of HDAC8) and tubastatin A (an inhibitor of HDAC6 and HDAC8) showed effects comparable to that of quisinostat (Supplementary Figure S3A). However, the selective HDAC6 inhibitor CAY10603 had no effect, suggesting a possible involvement of HDAC8 in the regulation of NADPH activity in PMA-stimulated THP-1 cells (Supplementary Figure S3A). This thesis is further supported by the finding that other HDAC inhibitors with little effects on HDAC8 (LMK-235, TMP269, and scriptaid) had no or only minor effect on NADPH oxidase activity (Supplementary Figure S3B).

### HAT Inhibition Decreases ROS Production in Human THP-1 Cells

**Figure 4:**
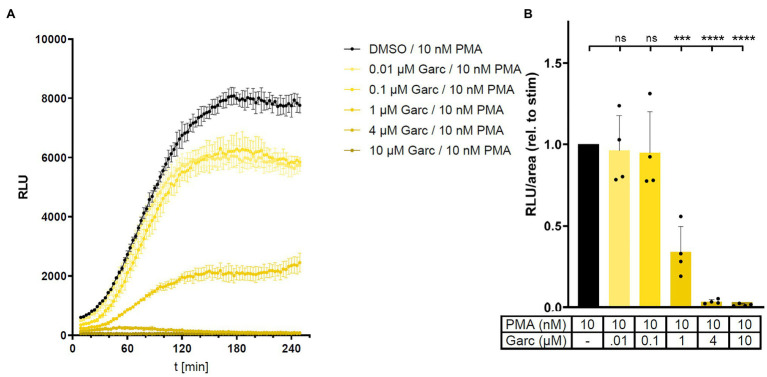
Histone acetyltransferase (HAT) inhibition reduces ROS production in human THP-1 cells. ROS production in PMA-stimulated human monocytic THP-1 cells was measured in the absence or presence of HAT inhibitor garcinol (Garc) in an L-012 assay. RLU were determined over time. Panel **(A)** shows results from a representative experiment, mean ± SD, *n* = 3. Panel **(B)** shows combined results from four independent experiments by calculating area under the curve in each experiment (relative to PMA alone). Columns represent mean ± SD. ^***^p < 0.001, ^****^p < 0.0001, compared with PMA, unpaired, two-tailed *t*-test.

To investigate the role of acetylation in the regulation of NADPH oxidase activity, we treated human THP-1 cells with histone acetyltransferase (HAT) inhibitors in the L-012 assay. Garcinol decreased PMA-stimulated ROS production in a concentration-dependent manner and was able to completely block L-012 signal at concentrations ≥ 4 μM (Figure 4). Similar findings were observed with two other HAT inhibitors, curcumin, and HAT Inhibitor II, with anacardic acid being an exception (Supplementary Figure S4). These results suggest that, in line with the effect of HDAC inhibitors, acetylation positively regulates NADPH oxidase activity.

### SIRT1 Directly Deacetylates Rac1 *in vitro*

**Figure 5:**
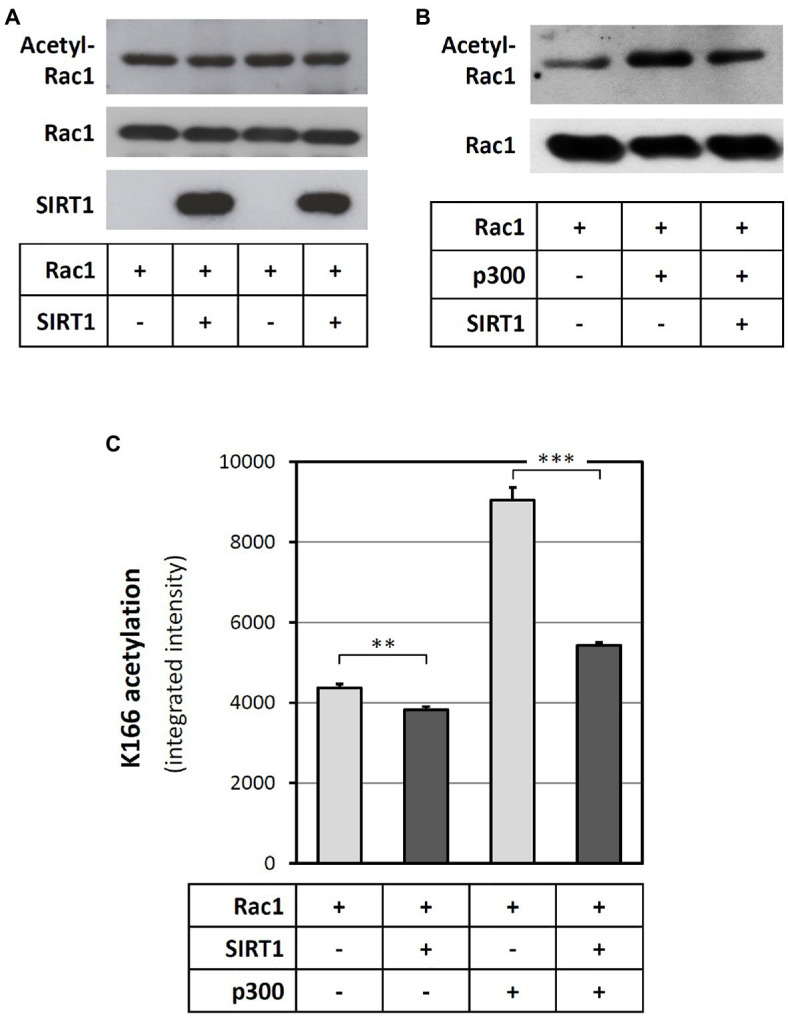
SIRT1 deacetylates Rac1 at the lysine 166 residue. Recombinant Rac1 protein was incubated with SIRT1 either directly **(A)** or after a pre-incubation with p300 acetyltransferase **(B,C)** in cell-free systems. Then, the reaction mixtures were subjected to SDS-PAGE and immunoblotted with antibodies against SIRT1, Rac1, and acetyl-lysine **(A,B)**, respectively. Panels **(A)** and **(B)** show representative blots. To explore which lysine residue was deacetylated by SIRT1, the reaction mixtures were subsequently digested with trypsin and analysed by mass spectrometry **(C)**. The columns represent mean ± SEM, *n* = 5. ^**^p < 0.01, ^***^p < 0.001, unpaired *t*-test.

As SIRT1 is a protein deacetylase, we hypothesized that SIRT1 may directly deacetylate Rac1. To verify this concept, recombinant human Rac1 was incubated *in vitro* with SIRT1. Then, the reaction mixture was subjected to SDS/PAGE. Rac1 acetylation was detected with an anti-acetyl-lysine antibody at the level of the Rac1 band. As shown in Figure 5A, a basal Rac1 acetylation could be found. Incubation of Rac1 with SIRT1 led to a reduction of Rac1 acetylation. In other words, SIRT1 could deacetylate Rac1. Incubation of Rac1 with acetyltransferase p300 markedly enhanced Rac1 acetylation, which could be significantly attenuated by SIRT1 (Figure 5B).

In order to identify which amino acid in the Rac1 molecule was deacetylated by SIRT1, we analyzed the samples with mass spectrometry. In this analysis, we observed a clear effect on lysine 166 (K166). Incubation of Rac1 with p300 led to a marked increase in K166 acetylation. SIRT1 significantly reduced the basal as well as the p300-induced K166 acetylation of Rac1 (Figure 5C). In one of the experiments, acetylation of K183 and K184 was also observed, but only in the presence of p300 (Supplementary Figure S5D).

### Rac1 K166 Deacetylation by SIRT1 Inhibits Rac1-p67phox Interaction

**Figure 6:**
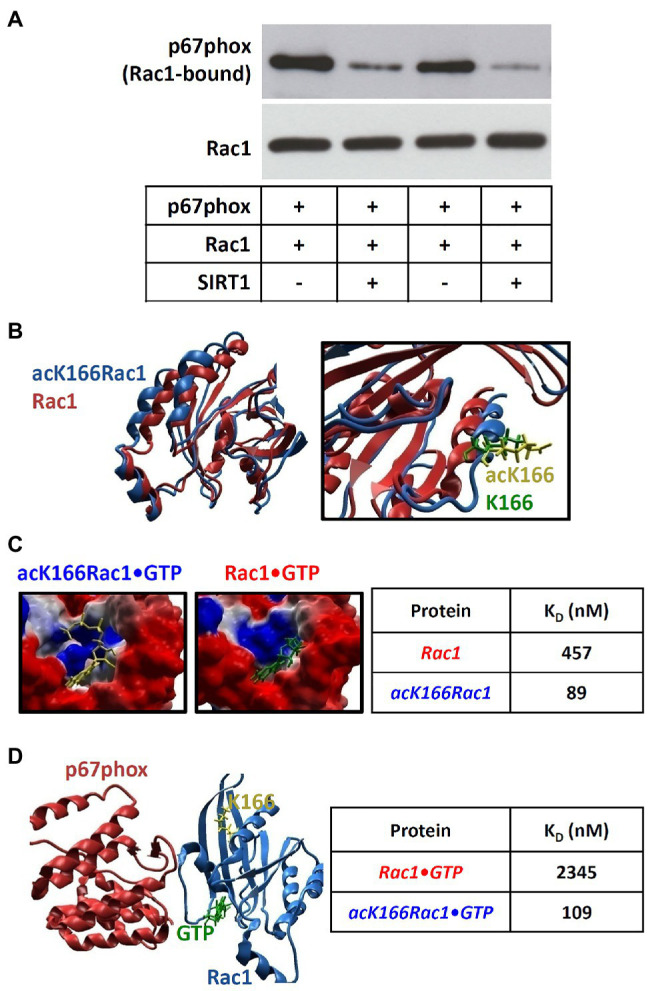
Rac1 deacetylation reduces its interaction with p67phox. **(A)**: Recombinant Rac1 protein was incubated with p67phox, with or without SIRT1, in cell-free systems. Then, Rac1 pull-down was performed with glutathione-agarose beads. Western blot analyses were performed with antibodies against Rac1 or p67phox using the Rac1-pulldown samples. Panel **(A)** shows representative blots. **(B)** Acetylation of lysine 166 (K166) on Rac1 leads to conformational changes as revealed by *in silico* modeling. **(C)** Conformational changes induced by acetylation of K166 on Rac1 enhance its binding affinity for GTP. **(D)** K166 acetylation enhances the binding activity of Rac1 to p67phox as calculated by computational modeling. K_D_, dissociation constant.

We then explored the functional consequence of Rac1 K166 deacetylation by SIRT1. When Rac1 recombinant protein (with basal lysine acetylation, see Figure 5A) was incubated with p67phox, a significant amount of p67phox could be detected in the Rac1-pulldown fraction (Figure 6A), confirming the interaction of Rac1 with p67phox. When SIRT1 was added to the reaction system, the amount of Rac1-bound p67phox was markedly reduced (Figure 6A).

To address how K166 deacetylation prevents Rac1-p67phox interaction, we performed computational modeling analyses. As shown in Figure 6B, acetylation of K166 results in a conformational change of Rac1. This acetylation-induced conformational change leads to a profound increase of the binding affinity of Rac1 for GTP (Figure 6C). The calculated dissociation constant (K_D_) of Rac1•GTP and acK166Rac1•GTP were 457 and 89 nM, respectively (Figure 6C). This is consistent with the previously reported K_D_ value (0.1 μM) of GTP binding to (acetylated) Rac1 (Haeusler et al., 2006). In other words, our results indicate that deacetylation of K166 reduces the binding affinity of Rac1 to GTP.

The interaction of Rac1 with the activator subunit p67phox is essential for the activity of NADPH oxidase enzyme complex. Our computational model predicts that deacetylation of K166 results in a marked inhibition of Rac1-p67phox interaction (Figure 6D). The calculated K_D_ values for Rac1-p67phox and acK166Rac1-p67phox were 2,345 and 109 nM, respectively (Figure 6D). This is in very good agreement with the previously reported K_D_ value (124 nM) of (acetylated) Rac1 binding to p67phox (Nisimoto et al., 1997). Notably, it has been shown that the binding of Rac1 to p67phox is GTP-dependent and it is only the GTP-bound form of Rac that interacts with p67phox (Diekmann et al., 1994). Thus, our computational results indicate that deacetylation of K166 leads to a 21.5-fold reduction of Rac1-p67phox affinity, which is consistent with the results from the *in vitro* experiment (Figure 6A).

## Discussion

In the present study, we show that NADPH oxidase-mediated superoxide production can be modified by acetylation and deacetylation. Both SIRT1 and non-sirtuin deacetylases play a role. SIRT1 can directly deacetylate Rac1 in a cell-free system. The downstream targets of HDCAs are still unknown.

The small GTPase Rac (Rac1 or Rac2) is a component of the NADPH oxidase complex (Drummond et al., 2011) and is essential for the activation of Nox1-3 (Bokoch and Zhao, 2006; Petry et al., 2010). The following discussion will focus on Nox2 for simplicity.

Nox2 is activated through a complex series of intracellular events (Brandes and Kreuzer, 2005; Bedard and Krause, 2007). Upon stimulation, the “organizer subunit” p47phox triggers the translocation of other cytosolic factors. Phosphorylated p47phox undergoes a conformational change, leading to its interaction with p22phox (*via* the SH3 domains of p47phox) and the cell membrane (*via* the PX domain of p47phox). The translocation of p47phox to the membrane further leads to the interaction of the “activator subunit” p67phox with Nox2, and also guides the small subunit p40phox to the complex. In parallel, Rac has affinity for the plasma membrane due to isoprenylation (Bokoch and Zhao, 2006) and is recruited to the membrane independent of p47phox (Heyworth et al., 1994). Nox2 is activated once the subunits are assembled, and starts to generate superoxide by electrons transferring from cytosolic NADPH to oxygen in the luminal or extracellular space (Bedard and Krause, 2007).

Recent studies demonstrate that Rac is not just an adapter protein; it has dual roles for Nox2 activation (Sarfstein et al., 2004). (i) Interaction between Rac and p67phox is essential for p67phox translocation to cell membrane (Petry et al., 2010). Rac recruits p67phox to the membrane independent of p47phox (Sarfstein et al., 2004; Miyano and Sumimoto, 2007). (ii) Rac is crucial for Nox2 activation. Constitutively expressed active Rac mutants can activate NADPH oxidase in COS^phox^ cells, even without any other stimulus (Price et al., 2002). Rac can interact with p67phox and induce an “activating” conformational change of p67phox, allowing the activation domain of p67phox to act on Nox2 (Sumimoto, 2008). This interaction between p67phox and Nox2 is indispensable for Nox2 activation (Lassegue et al., 2012). By binding to the dehydrogenase domain of Nox2 (Kao et al., 2008), the activation domain of p67phox promotes FAD reduction by Nox2 (Nisimoto et al., 1999) and electron flow from NADPH to flavin (Nisimoto et al., 1999), and thereby the activity of the NADPH oxidase complex. Thus, the interaction between Rac and p67phox is essential for Nox2 activation.

The N-terminal region of p67phox mainly consists of four tetratricopeptide repeat (TPR) motifs, followed by an activation domain, an SH3 domain, a PB1 domain, and at the C-terminus, a second SH3 domain (Bedard and Krause, 2007). The TPR motifs of p67phox are important for Rac binding (Koga et al., 1999; Lapouge et al., 2000), whereas the C-terminal SH3 domain can bind to the proline-rich region of p47phox (Bedard and Krause, 2007). The β-hairpin insertion in the TPR domain along with the loops connecting TPR1–TPR3, form the binding site for Rac (Lapouge et al., 2000; Sumimoto, 2008). With Rac/Rho chimeras, studies have shown that the N-terminal (residues 22–45) and C-terminal (residues 143–175) regions of Rac contributed to Rac/p67phox complex formation and NADPH oxidase activation (Diekmann et al., 1995). The structure of Rac/p67TPR shows that the residues Ser22, Thr25, Asn26, Phe28, Glu31, Gly30, Ala159, Leu160, and Gln162 in Rac represent the binding surface for p67phox (Lapouge et al., 2000). Although K166 is not a direct part of the interface, our computational modeling suggests that (de)acetylation of K166 has a compelling impact on the binding activity of Rac1 with p67phox. Rac2 is predominantly expressed in human neutrophils, while both Rac1 and Rac2 are present in monocytes and macrophages (Sumimoto, 2008). Rac 1 and Rac2 share the same amino acid residues in the interface of protein interactions. *In vitro* studies showed that binding affinities of the two Rac isoforms to p67phox were comparable (Lapouge et al., 2000).

Under normal conditions, Rac1 or Rac2 is bound to GDP in a complex with a member of the guanine nucleotide dissociation inhibitor (GDI) RhoGDI family. Guanine nucleotide exchange factors (GEF) control the activation of Rac1 or Rac2 through converting GDP to GTP (Petry et al., 2010). The Rac-p67phox interaction is in a GTP-dependent manner (Diekmann et al., 1994; Lapouge et al., 2000).

In the present study, we found that the affinity of Rac1 for GTP is regulated by acetylation of lysine 166 (K166) in Rac1. Our computational modeling results suggest that K166 acetylation affects the 3D structure of Rac1 leading to conformational changes in such a way that its binding affinity for GTP is increased (Figure 6C). This enhanced binding of GTP to K166-acetylated Rac1 shifts the equilibrium into the direction of acK166 Rac1•GTP enhancing the apparent affinity for p67phox binding by about 21.5-fold compared to non-acetylated Rac1 (Figure 6D). This computational prediction was confirmed by the *in vitro* binding study, which demonstrated that deacetylation of Rac1 by SIRT1 markedly decreases Rac1/p67phox binding (Figure 6A).

The original aim of the present study was to investigate the mechanism how resveratrol inhibits NADPH oxidase activity. Previous studies have shown that resveratrol can prevent and limit the progression of several diseases, such as cardiovascular disease, ischemic injuries, Alzheimer’s disease, and cancer. Besides, resveratrol has been identified as a caloric restriction mimetic, which shows properties of lifespan extending in various organisms (Baur and Sinclair, 2006; Agarwal and Baur, 2011). In the past, numerous direct or indirect target molecules have been identified to explain the versatile biological effects of resveratrol (Li et al., 2012). Among these molecules, the NAD^+^-dependent histone/protein deacetylase SIRT1 has received the most attention, because it has been postulated to mediate part of the effects of caloric restriction (Guarente, 2013) and has been considered a longevity gene.

An *in vitro* assay using a fluorogenic acetylated peptide derived from p53, a bona fide SIRT1 substrate, has suggested that resveratrol is a SIRT1 activator (Howitz et al., 2003). Later studies have questioned the claim of resveratrol as a direct SIRT1 activator. Recent studies have revealed that SIRT1 can be activated by resveratrol directly on certain peptide substrates [i.e., those with a large hydrophobic residue at position +1 or positions +1 and +6 (relative to the acetylated lysine); Hubbard et al., 2013]. In addition, it is likely that resveratrol activates SIRT1 indirectly *in vivo*, either by a pathway involving inhibition of phosphodiesterase enzymes and subsequent activation of AMPK (Park et al., 2012), or by promoting the binding of SIRT1 to its activator lamin A (Liu et al., 2012; Ghosh et al., 2013). The effects of resveratrol may be also partially attributed to an upregulation of SIRT1 expression *in vivo* (Csiszar et al., 2009; Xia et al., 2013).

In the cardiovascular system, SIRT1 can reduce oxidative stress through multiple mechanisms. (i) SIRT1 prevents the uncoupling of endothelial nitric oxide synthase (eNOS; Xia et al., 2010). Under pathological conditions, the deficiency of eNOS cofactor tetrahydrobiopterin (BH_4_) leads to eNOS uncoupling, and superoxide will be produced instead of NO (Forstermann and Munzel, 2006; Li and Forstermann, 2009, 2013). We have previously shown that resveratrol can prevent the uncoupling of eNOS in ApoE-KO mice by upregulating GTP cyclohydrolase 1 (GCH1), the rate-limiting enzyme in BH_4_ *de novo* biosynthesis (Xia et al., 2010). This effect of resveratrol in upregulating GCH1 is mediated by SIRT1, as demonstrated in endothelial cells *in vitro* (Xia et al., 2010). (ii) SIRT1 can stimulate mitochondrial biogenesis and attenuate mitochondrial ROS generation (Csiszar et al., 2009). SIRT1 protects mitochondrial function by deacetylating it downstream target PGC-1α (Lagouge et al., 2006). (iii) SIRT1 enhances the expression of SOD, the enzyme which is responsible for catalyzing the dismutation of superoxide into hydrogen peroxide. This has been shown for the copper/zinc SOD (SOD1; Spanier et al., 2009; Xia et al., 2010) and the mitochondrial manganese SOD (SOD2; Tanno et al., 2010). SIRT1 upregulates SOD2 at least partially *via* FOXO1 (Hsu et al., 2010). (iv) SIRT1 also stimulates the expression of glutathione peroxidase 1 (GPx1; Spanier et al., 2009; Xia et al., 2010) and catalase (Alcendor et al., 2007) in cardiovascular tissues, accelerating the inactivation of hydrogen peroxide and peroxynitrite.

The present study provides a novel mechanism as to how SIRT1 decreases oxidative stress in the cardiovascular system – deacetylation of Rac1 at K166 (Figure 5). This modification of Rac1 reduced Rac1-p67phox interaction, which was evident *in vitro* using cell-free systems (Figure 6). The reduced activity of membrane NADPH oxidase by resveratrol (Figure 1) could be a consequence of Rac1 deacetylation by SIRT1, although direct evidence is still missing.

Until now, Rac1 is known to be subject to various post-translational modifications, including ubiquitination, palmitoylation, phosphorylation, geranylgeranylation, and SUMOylation (Murali and Rajalingam, 2014). The present study shows that Rac1 can be also acetylated. Potential biological consequences (in addition to Rac1-phox67 interaction) of this post-translational modification are beyond the scope of the present study. Interestingly, the acetylation site K166 is an ubiquitination site of Rac1 (Murali and Rajalingam, 2014). Thus, acetylation of K166 might prevent Rac1 ubiquitination and subsequent proteasomal degradation. However, this currently remains solely speculative and further studies are warranted.

Our study has some limitations. First, the dissociation constants for Rac1-p67phox and acK166Rac1-p67phox were calculated by computational modeling analyses. Thus, the values should be considered as theoretical numbers, and need to be verified in future studies with quantitative methods. Nevertheless, the prediction of the computational modeling analyses was supported by our *in vitro* binding study demonstrating that deacetylation of Rac1 by SIRT1 markedly decreases Rac1/p67phox binding (Figure 6A). Second, experimental confirmation of the impact of K166 acetylation on Rac1-p67phox interaction or on NADPH oxidase activity is still missing. This should be done in future studies with K166 mutant Rac1 forms. Third, we have identified K166 (and K183/184) as the acetylation sites in an *in vitro* assay using recombinant proteins. We have yet no evidence on whether K166 (de)acetylation also occurs *in vivo* or whether K166 is the main lysine residue subjected to acetylation. It would be remarkably interesting to perform Rac1 acetylation analyses using samples from experimental animals or human subjects, both under healthy and diseased conditions, to find out the *in vivo* significance of Rac1 acetylation regulation. Fourth, although we have provided evidence that HDAC and HAT regulate the activity of NADPH oxidase, the detailed mechanisms are still not known. The (de)acetylation targets of HDAC and HAT may be some NADPH oxidase subunits (e.g., Rac). However, it is also possible that NADPH oxidase activity is regulated by some other proteins that are targeted by HDAC and HAT. In such cases, acetylation and deacetylation would represent indirect mechanisms regulating NADPH oxidase activity.

In conclusion, the present study demonstrates that NADPH oxidase-mediated superoxide production is regulated by both SIRT1 and non-sirtuin deacetylases. Rac1 may be a direct molecular target of SIRT1, at least in cell-free systems. Deacetylation of Rac1 at the lysine 166 residue by SIRT1 decreases its affinity for GTP and thereby prevents its interaction with p67phox *in vitro*. This may represent a novel mechanism regulating NADPH oxidase activity. However, evidence for a causal role of K166 acetylation in this regulation is still missing. The downstream target(s) of HDACs remain to be identified. Further studies are warranted to investigate the significance of NADPH oxidase activity regulation by acetylation and deacetylation mechanisms *in vivo*.

## Data Availability Statement

The raw data supporting the conclusions of this article will be made available by the authors, without undue reservation.

## Ethics Statement

The animal study was reviewed and approved by Landesuntersuchungsamt Rheinland-Pfalz.

## Author Contributions

NX conceived the study, performed experiments, analyzed and interpreted data, and wrote the manuscript. ST, OL, MK, AD, and GR performed experiments, analyzed and interpreted data, and reviewed and edited the manuscript. TS, AD, TM, and UF contributed to discussion and reviewed and edited the manuscript. HL conceived the study and wrote the manuscript. All authors contributed to the article and approved the submitted version.

## Conflict of Interest

The authors declare that the research was conducted in the absence of any commercial or financial relationships that could be construed as a potential conflict of interest.

## Publisher’s Note

All claims expressed in this article are solely those of the authors and do not necessarily represent those of their affiliated organizations, or those of the publisher, the editors and the reviewers. Any product that may be evaluated in this article, or claim that may be made by its manufacturer, is not guaranteed or endorsed by the publisher.
