# Supplementary material for: Regulation of NADPH Oxidase-Mediated Superoxide Production by Acetylation and Deacetylation
Source: Front Physiol. 2021 Aug 12;12:693702. doi: 10.3389/fphys.2021.693702 (PMC8387964; doi:10.3389/fphys.2021.693702)
Supplement: Supplementary file 1 [file Data_Sheet_1.pdf]

Data supplement to

DOI: 10.3389/fphys.2021.693702

Regulation of NADPH oxidase-mediated superoxide production by acetylation and deacetylation

Ning Xia[1], Stefan Tenzer[2], Oleg Lunov[3,4], Martin Karl[1], Thomas Simmet[3], Andreas Daiber[5], Thomas Münzel[5], Gisela Reifenberg[1], Ulrich Förstermann[1], Huige Li[1,*]

[1]Department of Pharmacology, Johannes Gutenberg University Medical Center, Mainz, Germany;
[2]Department of Immunology, Johannes Gutenberg University Medical Center, Mainz, Germany;
[3]Institute of Pharmacology of Natural Products and Clinical Pharmacology, Ulm University, Ulm, Germany;
[4]Department of Optical and Biophysical Systems, Institute of Physics ASCR, Prague, Czech Republic;
[5]Department of Cardiology, Cardiology I, Johannes Gutenberg University Medical Center, Mainz, Germany

Running title: NADPH oxidase & acetylation

* **Correspondence** and requests for materials should be addressed to
Prof. Dr. Huige Li
Department of Pharmacology
Johannes Gutenberg University Medical Center
Langenbeckstr. 1, 55131 Mainz, Germany
Telefon: +49-6131-17 9348
Telefax: +49-6131-17 9329
E-mail: huigeli@uni-mainz.de

Animals and treatment.

Male ApoE-KO mice (Charles River Laboratories, Sulzfeld, Germany) aged six months were used. Resveratrol (trans-3,4',-5-trihydroxystilebene) was obtained from Cayman Chemical (Ann Arbor, Michigan, USA). Mice were treated with resveratrol at doses of 30 or 100 mg/kg via gavage for seven days as previously described (Xia et al., 2010) and then killed with an overdose of pentobarbital. The heart was taken for protein analyses, membrane translocation or measurement of NADPH oxidase activity. All animal experiments were performed in accordance with the German animal protection law and the guidelines for the use of experimental animals as stipulated by the Guide for the Care and Use of Laboratory Animals of the National Institutes of Health.

NADPH oxidase activity assay with lucigenin chemiluminescence.

A modified version of the previously (Daiber et al., 2004) described method for measurement of membrane NADPH oxidase activity was used. Briefly, heart tissue was homogenized (glass/glass) in homogenization buffer containing 50 mM Tris•HCl, pH 7.4, 2 mM dithiothreitol, and a protease inhibitor cocktail (Roche) for general use and centrifuged at 2,000 g for 5 min at room temperature. The supernatant was removed and centrifuged at 20,000 g for 20 min at 4°C. Again, the supernatant was removed and centrifuged at 100,000 g for 60 min at 4°C. The pellet was resuspended in homogenization buffer (without dithiothreitol) and for the assay further diluted in PBS to give a final protein concentration of 0.2 mg/ml. The lucigenin (5 µM)-derived chemiluminescence of the membrane suspensions was detected in a Lumat LB 9507 (Berthold) in the presence of 200 µM NADPH.

Culture of THP-1 cells and ROS measurement with L-012

THP-1 cells (Qin, 2012) were maintained in RPMI 1640 Medium supplemented with GlutaMAX™ (Gibco/ Thermo Fisher Scientific), 10% FCS and 1% penicillin/streptomycin.

For luminescence-based ROS detection, THP-1 cells were pre-incubated with 100 µM L-012 (FUJIFILM Wako Chemicals) in pre-warmed Hank's buffered solution at a concentration of $0.5x10^6$ cells/ml for 5 min at 37°C. Subsequently, the cells were incubated for 15 min at 37°C with HDAC/HAT inhibitors (or DMSO as solvent control) before 10 nM phorbol 12-myristate 13-acetate (PMA, Sigma-Aldrich; or DMSO as solvent control) was added. 100.000 cells/well were transferred immediately to a luminescence-adequate 96-well plate and luminescence was determined with a microplate reader (SpectraMax® iD3, Molecular Devices).

***In vitro* acetylation and deacetylation of Rac1.**

One µg GST-tagged recombinant Rac1 (Cytoskeleton Inc., Denver, USA) was incubated with acetyl-CoA (20 µM, Active Motif) and p300 acetyltransferase (100 ng, Active Motif) at 30°C for 30 min. The acetylation reaction was immediately followed by the deacetylation reaction by adding deacetylation buffer (25 mM Tris•HCl, pH 8.0, 137 mM NaCl, 2.7 mM KCl, 1 mM $MgCl_2$/1 mg/ml BSA), 1 mM NAD^+, and the active recombinant SIRT1 (100 units; Biozol, MBL Inc., Woburn) and incubating at 37°C for 1 h. The reaction mixture was used for subsequent immunoblotting, pull-down or mass spectrometry analyses. In case for immunoblotting, the reaction mixtures were subjected to SDS/PAGE and immunoblotted with antibodies against SIRT1 (Santa Cruz), Rac1 (Millipore), p67phox (Epitomics) or acetylated lysine (Cell Signaling).

In-gel digest and mass spectrometry.

The Rac1 (de)acetylation reaction mixtures (see paper) were subjected to SDS/PAGE and then stained with Coomassie blue R-250 (Sigma), and the relevant protein bands excised and sliced into small pieces. After destaining and drying, gel slices were reduced with 2 mM DTT at 55 °C and alkylated with 15 mM iodacetamide at room temperature in the dark for 1 h each. After washing and drying, trypsin

digests were done at 37 °C overnight (0.2 µg of trypsin per gel slice). The resulting peptides were transferred into an autosampler vial for peptide analysis via LCMS.

UPLC configuration

Capillary liquid chromatography of tryptic peptides was performed with a Waters NanoAcquity UPLC system equipped with a 75 µm x 150 mm BEH C18 reversed phase column and a 2.6 µl PEEKSIL-sample loop (SGE, Darmstadt, Germany) as described before(Tenzer et al., 2011).

Mass spectrometer configuration

Mass spectrometry analysis of tryptic peptides was performed using a Waters Q-TOF Premier API system, operated in V-mode with typical resolving power of R = 10.000. All analyses were performed using positive mode ESI using a NanoLockSpray source as described before(Wigand et al., 2009). For fragment identification, the instrument was run in data-directed acquisition mode, selecting the three most intense peaks for MS-MS fragmentation analysis. Fragmentation of the parent ion was achieved by collision with argon atoms. Collision energy was varied from 15 to 40 eV dependent on precursor ion mass and charge. The integration time for the TOF analyzer was 1 s with an interscan delay of 0.1 s.

Data processing and protein identification

The liquid chromatography tandem MS (LCMSMS) data were processed and searched by using PROTEINLYNX GLOBAL SERVER, Ver. 2.4. (Waters). Protein identifications were assigned by searching a custom compiled database containing human proteins (Uniprot Swissprot Release 2011-08, 20256 entries) supplemented with known possible contaminants (trypsin, GST) with the precursor and fragmentation data afforded by the LC-MSMS acquisition method. The mass error tolerance values were typically <5 ppm. Peptide identifications were restricted to tryptic peptides with no more than two missed cleavages and fixed cysteine carbamidomethylation, allowing for methionine oxidation, lysine acetylation and glutamine/asparagine deamidation as variable modifications. All identified peptide sequences were verified by manual interpretation of the fragment spectra.

***In vitro* Rac1-p67phox interaction and Rac1 pull-down experiment.**

The Rac1 deacetylation reaction mixtures (Rac1 ± SIRT1) were incubated with 3 μg recombinant p67phox (OriGene) at 4°C for 1 h. Then, Rac1 pull-down was performed with 40μl 50% glutathione-agarose beads (BD Biosciences), as Rac1 was the only protein carrying a GST-tag in the reaction mixture. After washing, the pull-down complexes were subjected to SDS/PAGE and immunoblotted with anti-Rac1 (Millipore), anti-p67phox (BD Biosciences) and anti-acetylated lysine (Cell Signaling).

Computational modelling.

Computational docking and scoring studies of the interaction of Rac1 and acK166Rac1with p67phox were performed using Hex 6.3 (Ritchie et al., 2008). Modelling of GTP binding to Rac1 and acK166Rac1 was done using Molegro Virtual Docker 5 (Thomsen and Christensen, 2006). The original parameters of blind docking were used in combination with an evaluation algorithm based on binding free energy (ΔG) (Hetenyi and van der Spoel, 2002). Structures of Rac1 and p67phox were from the Protein Data Bank (1E96) (Lapouge et al., 2000). A molecular model of acK166Rac1 was generated by template LOMETS homology modeling with structure fragmentation and reassembling by replica-exchange Monte Carlo simulations using the I-TASSER standalone package (version 1.1) (Zhang, 2008;2009;Roy et al., 2010). Three-dimensional models of Rac1-p67phox and acK166Rac1-p67phox complexes with surface charge distribution were created with Molegro Virtual Docker 5 (Thomsen and Christensen, 2006).

References

Daiber, A., August, M., Baldus, S., Wendt, M., Oelze, M., Sydow, K., Kleschyov, A.L., and Munzel, T. (2004). Measurement of NAD(P)H oxidase-derived superoxide with the luminol analogue L-012. *Free radical biology & medicine* 36**,** 101-111.

Hetenyi, C., and Van Der Spoel, D. (2002). Efficient docking of peptides to proteins without prior knowledge of the binding site. *Protein science : a publication of the Protein Society* 11**,** 1729-1737.

Lapouge, K., Smith, S.J., Walker, P.A., Gamblin, S.J., Smerdon, S.J., and Rittinger, K. (2000). Structure of the TPR domain of p67phox in complex with Rac.GTP. *Molecular cell* 6**,** 899-907.

Qin, Z. (2012). The use of THP-1 cells as a model for mimicking the function and regulation of monocytes and macrophages in the vasculature. *Atherosclerosis* 221**,** 2-11.

Ritchie, D.W., Kozakov, D., and Vajda, S. (2008). Accelerating and focusing protein-protein docking correlations using multi-dimensional rotational FFT generating functions. *Bioinformatics* 24**,** 1865-1873.

Roy, A., Kucukural, A., and Zhang, Y. (2010). I-TASSER: a unified platform for automated protein structure and function prediction. *Nature protocols* 5**,** 725-738.

Tenzer, S., Docter, D., Rosfa, S., Wlodarski, A., Kuharev, J., Rekik, A., Knauer, S.K., Bantz, C., Nawroth, T., Bier, C., Sirirattanapan, J., Mann, W., Treuel, L., Zellner, R., Maskos, M., Schild, H., and Stauber, R.H. (2011). Nanoparticle size is a critical physicochemical determinant of the human blood plasma corona: a comprehensive quantitative proteomic analysis. *ACS Nano* 5**,** 7155-7167.

Thomsen, R., and Christensen, M.H. (2006). MolDock: a new technique for high-accuracy molecular docking. *Journal of medicinal chemistry* 49**,** 3315-3321.

Wigand, P., Tenzer, S., Schild, H., and Decker, H. (2009). Analysis of protein composition of red wine in comparison with rose and white wines by electrophoresis and high-pressure liquid chromatography-mass spectrometry (HPLC-MS). *Journal of agricultural and food chemistry* 57**,** 4328-4333.

Xia, N., Daiber, A., Habermeier, A., Closs, E.I., Thum, T., Spanier, G., Lu, Q., Oelze, M., Torzewski, M., Lackner, K.J., Munzel, T., Forstermann, U., and Li, H. (2010). Resveratrol reverses endothelial nitric-oxide synthase uncoupling in apolipoprotein E knockout mice. *The Journal of pharmacology and experimental therapeutics* 335**,** 149-154.

Zhang, Y. (2008). I-TASSER server for protein 3D structure prediction. *BMC bioinformatics* 9**,** 40.

Zhang, Y. (2009). I-TASSER: fully automated protein structure prediction in CASP8. *Proteins* 77 Suppl 9**,** 100-113.

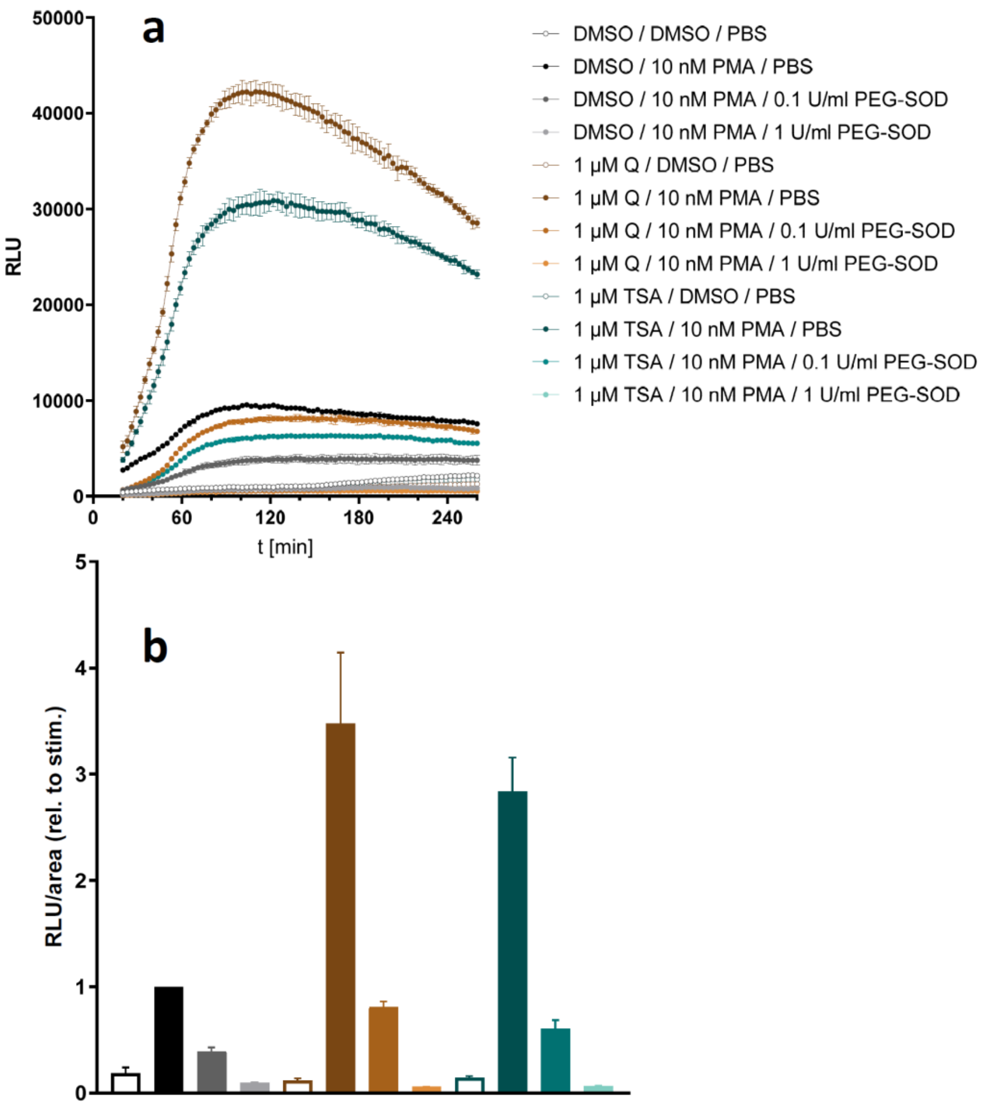

PEG-SOD	-	-	.1	1	-	-	.1	1	-	-	.1	1
PMA	-	+	+	+	-	+	+	+	-	+	+	+
Q	-	-	-	-	+	+	+	+	-	-	-	-
TSA	-	-	-	-	-	-	-	-	+	+	+	+

Fig. S1. L-012 signal in THP-1 cells is sensitive to PEG-SOD quenching. ROS production in PMA-stimulated human monocytic THP-1 cells was measured in the absence or presence of HDAC inhibitors quisinostat (Q, 1 µM) or trichostatin A (TSA, 1 µM) in an L-012 assay. PEG-SOD concentration-dependently decreases the relative light units (RLU) over time. Panel **a** shows a representative experiment (mean ± SD, n=3). Panel **b** shows combined results from 3 independent experiments by calculating area under the curve in each experiment (relative to PMA alone). Columns represent mean ± SD.

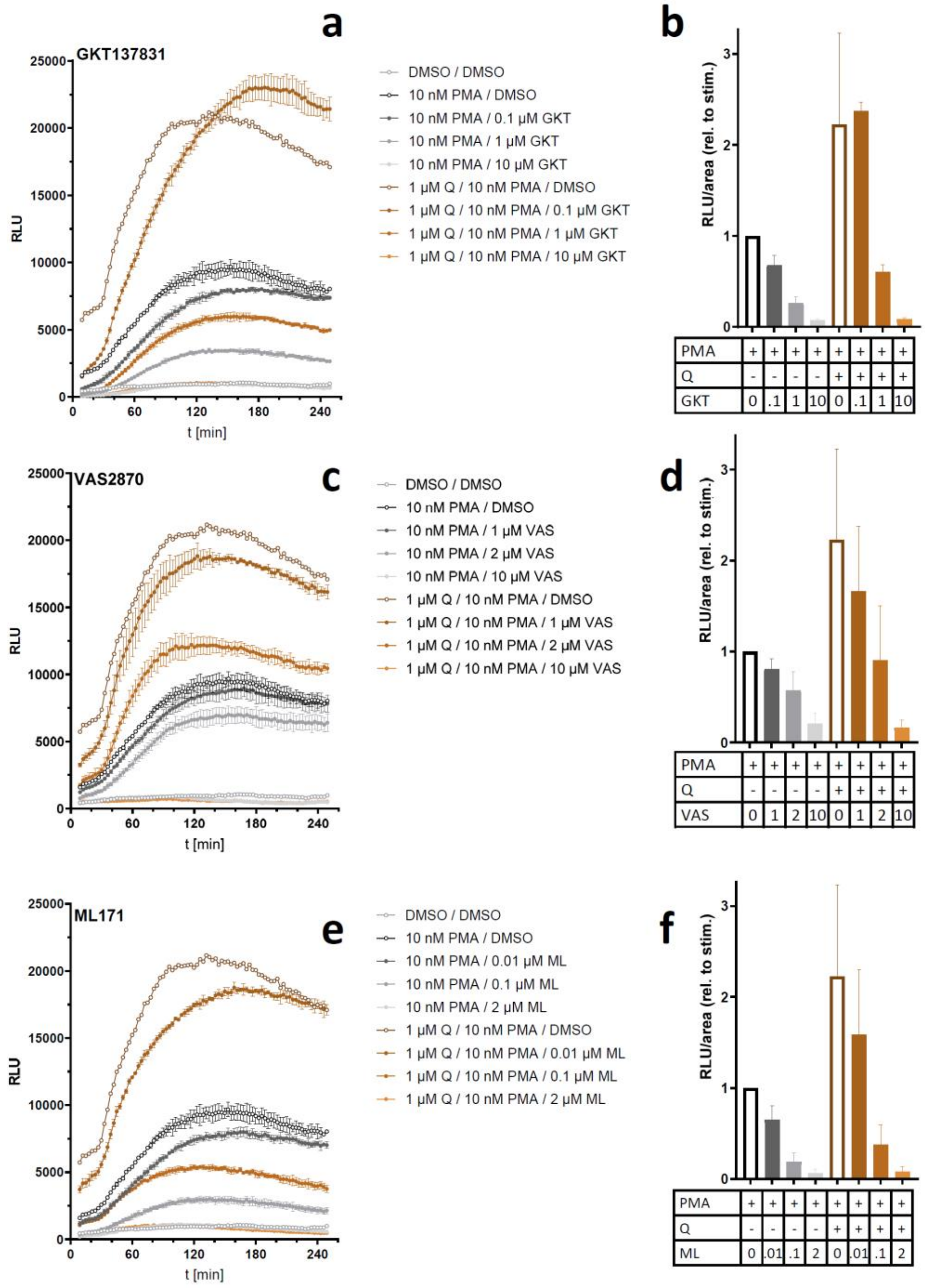

Fig. S2. ROS production by THP-1 cells can be prevented by inhibitors of NADPH oxidase in a concentration-dependent manner. ROS production in PMA-stimulated human monocytic THP-1 cells was measured in the absence or presence of 1 µM quisinostat (Q) in an L-012 assay. NAPDH oxidase inhibitors GKT137831, VAS2870 and ML171 concentration-dependently decrease the relative light units (RLU) over time. Panels **a**, **c** and **e** show results from representative experiments; mean ± SD, n=3 each. Panels **b**, **d** and **f** show combined results from 2-4 independent experiments by calculating area under the curve in each experiment (relative to PMA alone). Columns represent mean ± SD.

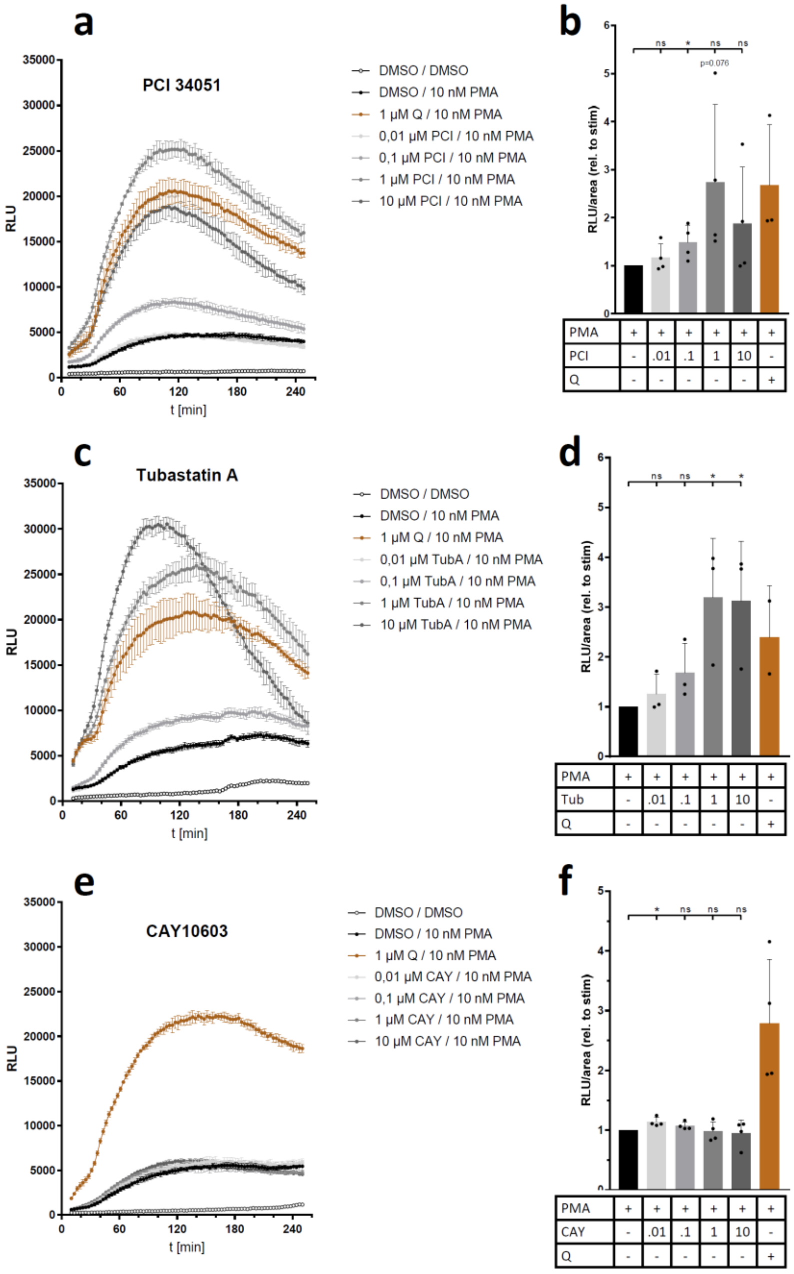

Fig. S3A. Inhibitors of HDAC8 enhances ROS production by THP-1 cells. ROS production in PMA-stimulated human monocytic THP-1 cells was measured in an L-012 assay in the absence or presence of PCI34051 (an inhibitor of HDAC8), tubastatin A (inhibits HDAC6, but also HDAC8) or CAY10603 (a selective HDAC6 inhibitor). Quisinostat (Q, 1 µM) was used as a positive control. Relative light units (RLU) were determined over time. The left panels (**a**, **c**, **e**) show representative experiments with n=3 each, mean ± SD. The right panels (**b**, **d**, **f**) show combined results from 3-4 independent experiments by calculating the area under the curve (relative to PMA). Columns represent mean ± SD. *P<0.05, compared with PMA, unpaired, two-tailed t test.

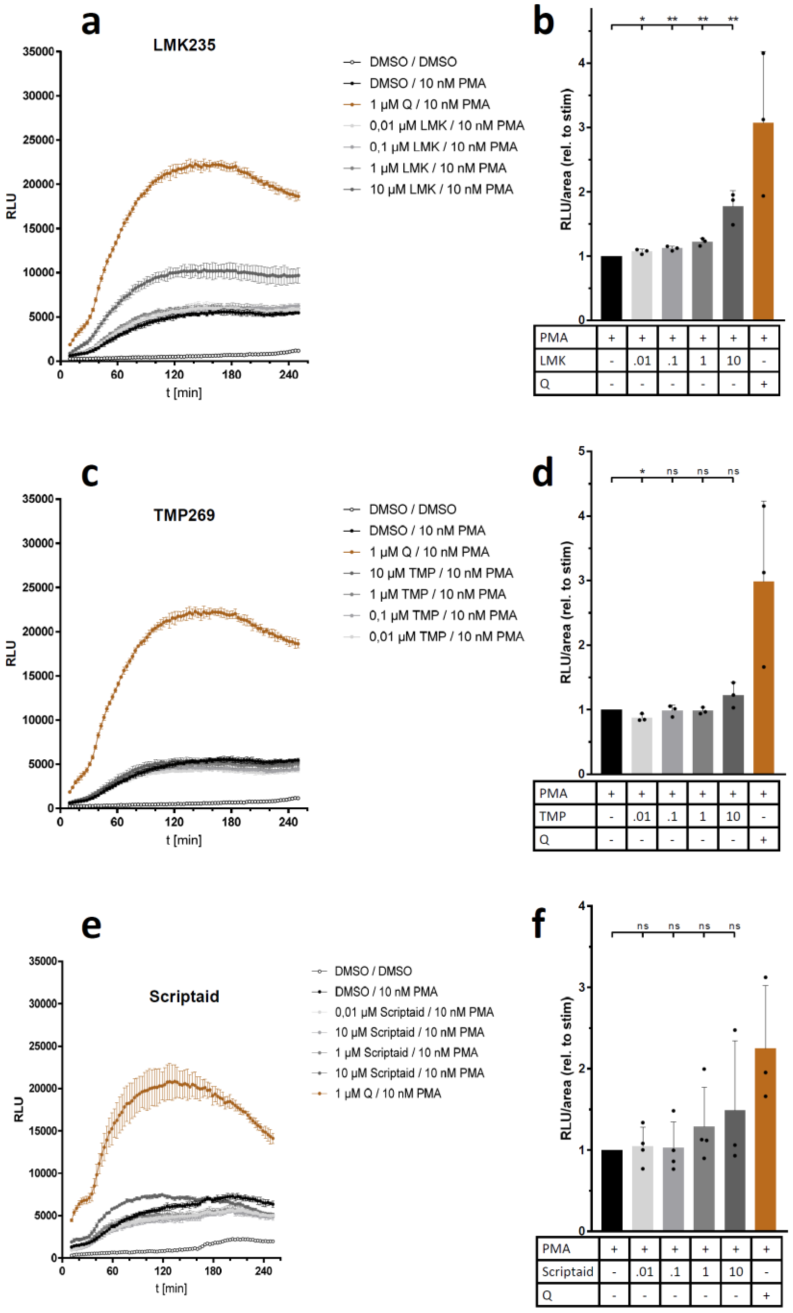

Fig. S3B. Non-HDAC8 inhibitors had little effects on ROS production by THP-1 cells. ROS production in PMA-stimulated human monocytic THP-1 cells was measured in an L-012 assay in the absence or presence of HDAC inhibitors including LMK235 (a selective inhibitor of HDAC4 and HDAC5), TMP269 (a selective inhibitor of HDAC4, HDAC5, HDAC7 and HDAC9) and scriptaid (IC_{50} for HDAC8: 6.81 µM). Quisinostat (Q, 1 µM) was used as positive control. The left panels (**a**, **c**, **e**) show representative experiments with n=3 each, mean ± SD. The right panels (**b**, **d**, **f**) show combined results from 3-4 independent experiments by calculating the area under the curve (relative to PMA). Columns represent mean ± SD. *P<0.05, **P<0.01, compared with PMA, unpaired, two-tailed t test.

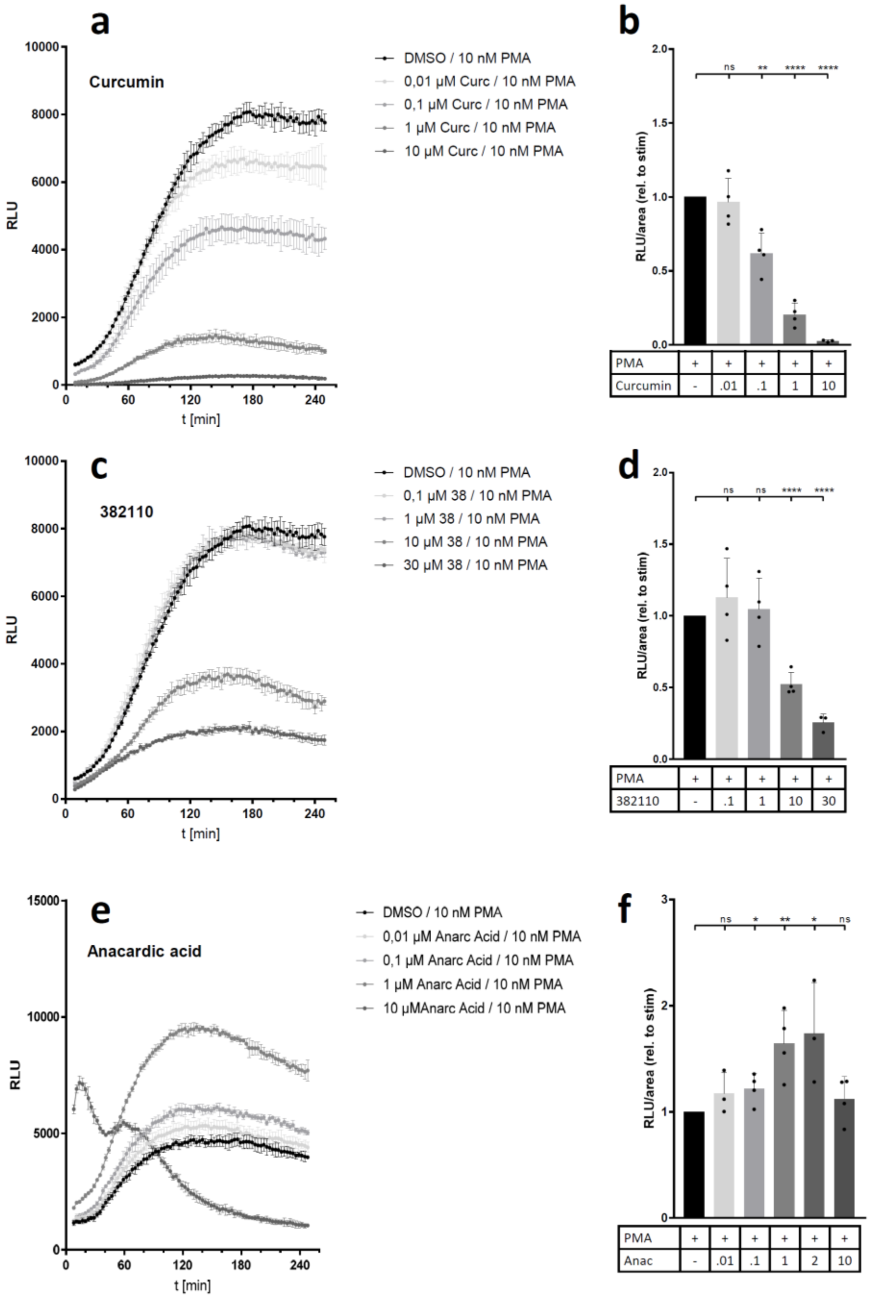

Fig. S4. HAT inhibitors decrease ROS production by THP-1 cells in a concentration-dependent manner. ROS production in PMA-stimulated human monocytic THP-1 cells was measured in an L-012 assay in the absence or presence of histone acetyl-transferase (HAT) inhibitors including curcumin, HAT Inhibitor II (Sigma 382110), and anacardic acid. The left panels (**a**, **c**, **e**) show representative experiments with n=3 each, mean ± SD. The right panels (**b**, **d**, **f**) show combined results from 3-4 independent experiments by calculating the area under the curve (relative to PMA). Columns represent mean ± SD. *P<0.05, **P<0.01, ****P<0.0001, compared with PMA, unpaired, two-tailed t test.

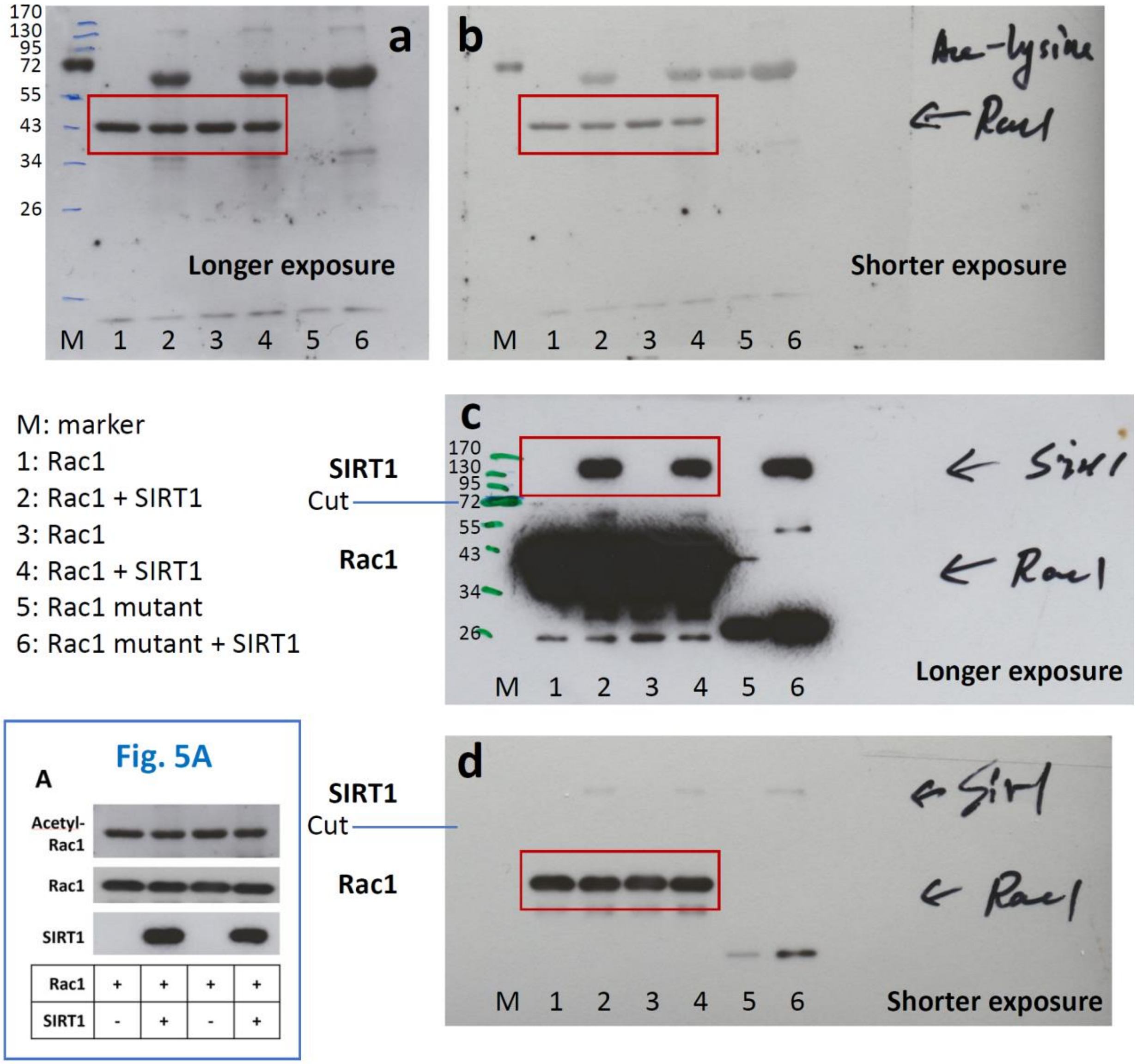

Fig. S5A. Rac1 deacetylation by SIRT1 in a cell-free system (for the main figure Fig. 5A).
The recombinant Rac1 protein (GST-tagged, lanes 1-4) was incubated with the active recombinant SIRT1 protein (lanes 2, 4, 6). In lanes 5 and 6, a mutant Rac1 (Q61L; histidine-tagged) was used. The reaction mixture was subjected to SDS/PAGE and transferred to nitrocellulose membranes.
(a) and **(b)**: The membrane was immunoblotted with an antibody against acetyl-lysine.
(c) and **(d)**: The membrane was cut into two parts, with the upper part being blotted with an antibody against SIRT1 and the lower part blotted with an antibody against Rac1.
The red boxes indicate regions of interest for the main figure (Fig. 5A)

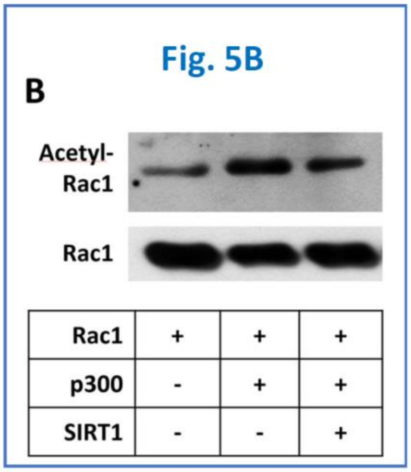

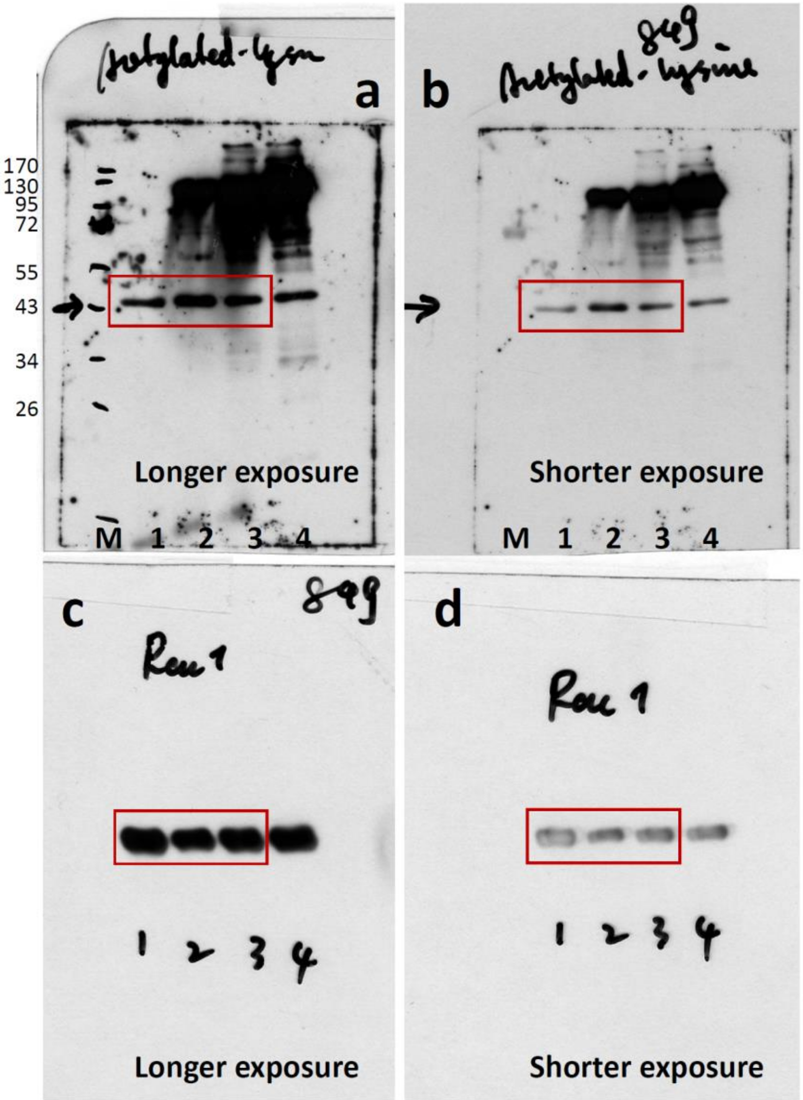

	1	2	3	4
Rac1	+	+	+	+
p300	-	+	+	+
SIRT1	-	-	+	+

Fig. S5B. Rac1 deacetylation by SIRT1 in a cell-free system (for the main figure Fig. 5B).

The recombinant Rac1 protein (GST-tagged) was incubated with the p300 acetyl-transferase followed by the deacetylation reaction by the active recombinant SIRT1. The reaction mixture was subjected to SDS/PAGE and transferred to nitrocellulose membranes.

(a) and **(b)**: The membrane was immunoblotted with an antibody against acetyl-lysine.

(c) and **(d)**: The membrane was blotted with an antibody against Rac1.

The red boxes indicate regions of interest for the main figure (Fig. 5B)

Peptide 164-174; sequence: GLK(Ac)TVFDEAIR
m/z 645.86; delta m/z = -0.9ppm

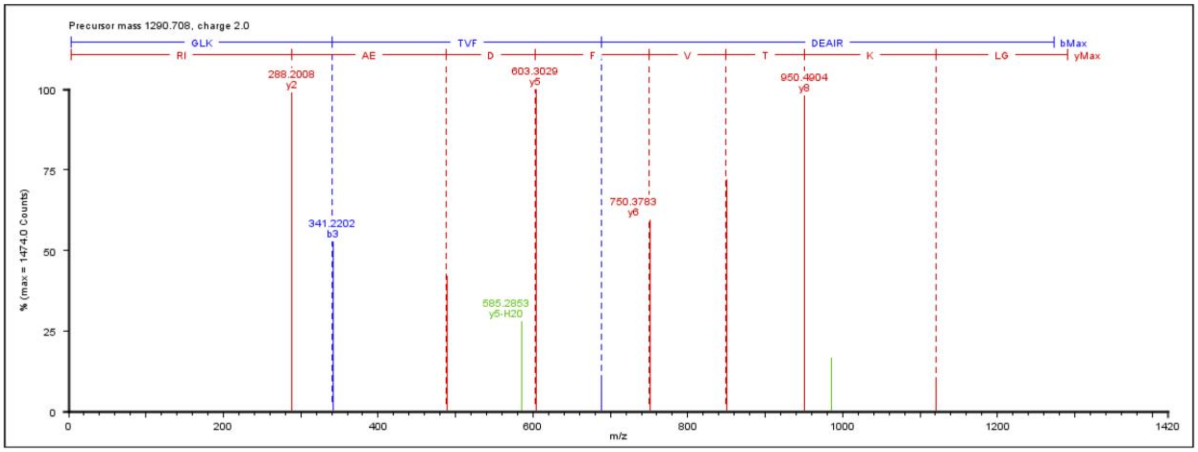

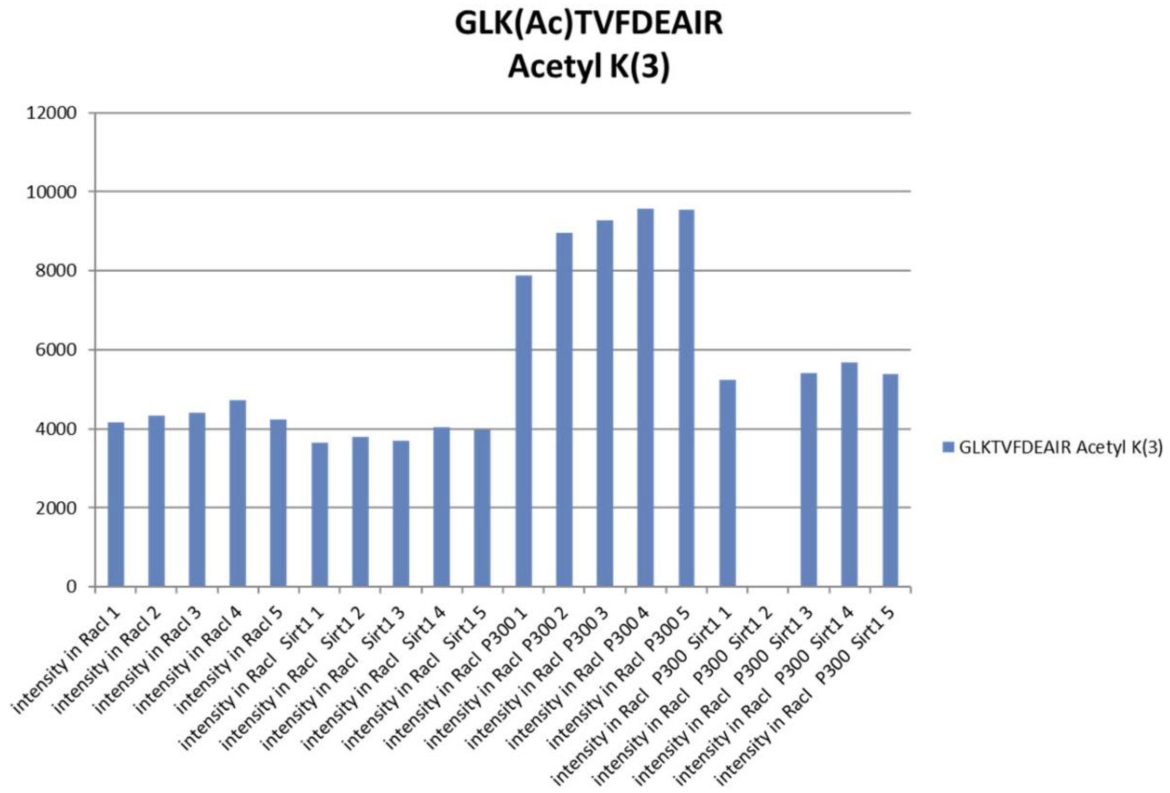

Fig. S5C. Rac1 acetylation at K166 detected with mass spectrometry (supporting data for the main figure Fig. 5C).

Peptide 175-185, sequence: AVLCPPPVK(Ac)K(Ac)R
m/z 1348.778, delta m/z = +0.53 ppm

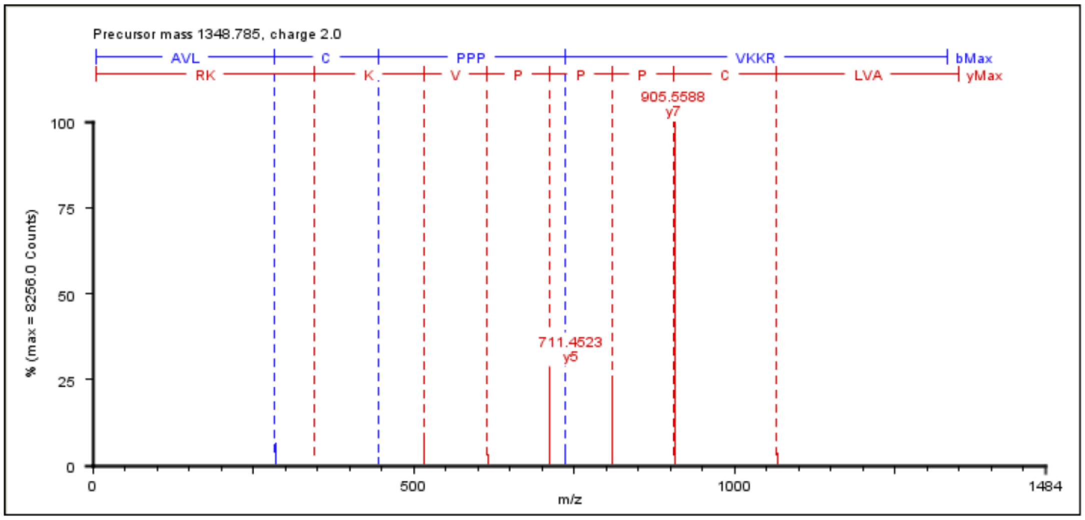

AVLCPPPVK(Ac)K(Ac)R
Carbamidomethyl C(4), Acetyl K(9), Acetyl K(10)

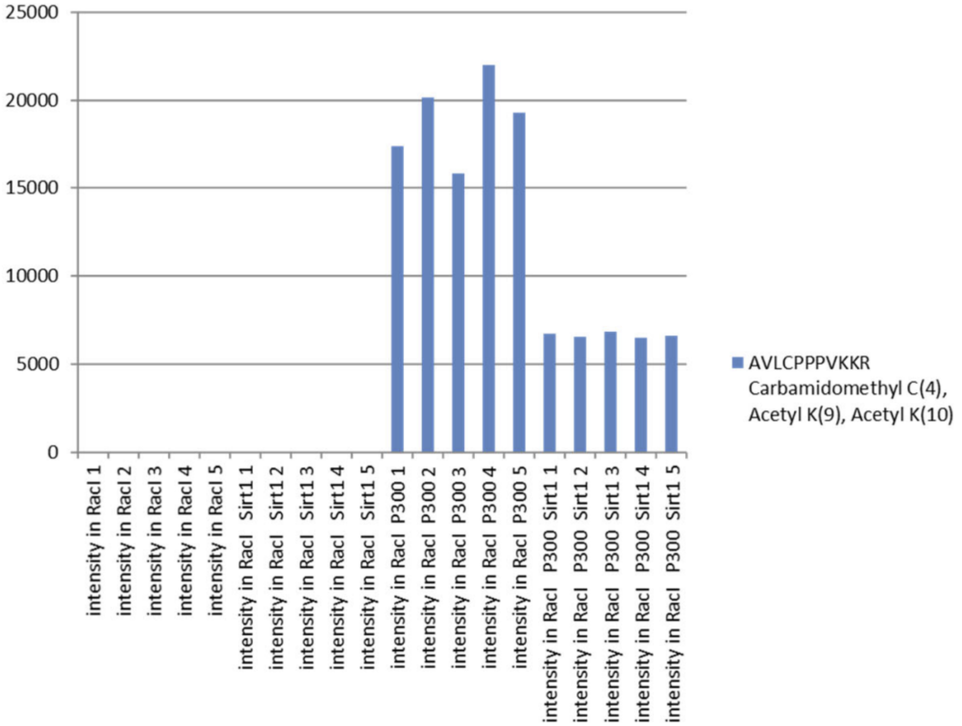

Fig. S5D. Rac1 acetylation at K183 and K184 detected with mass spectrometry.

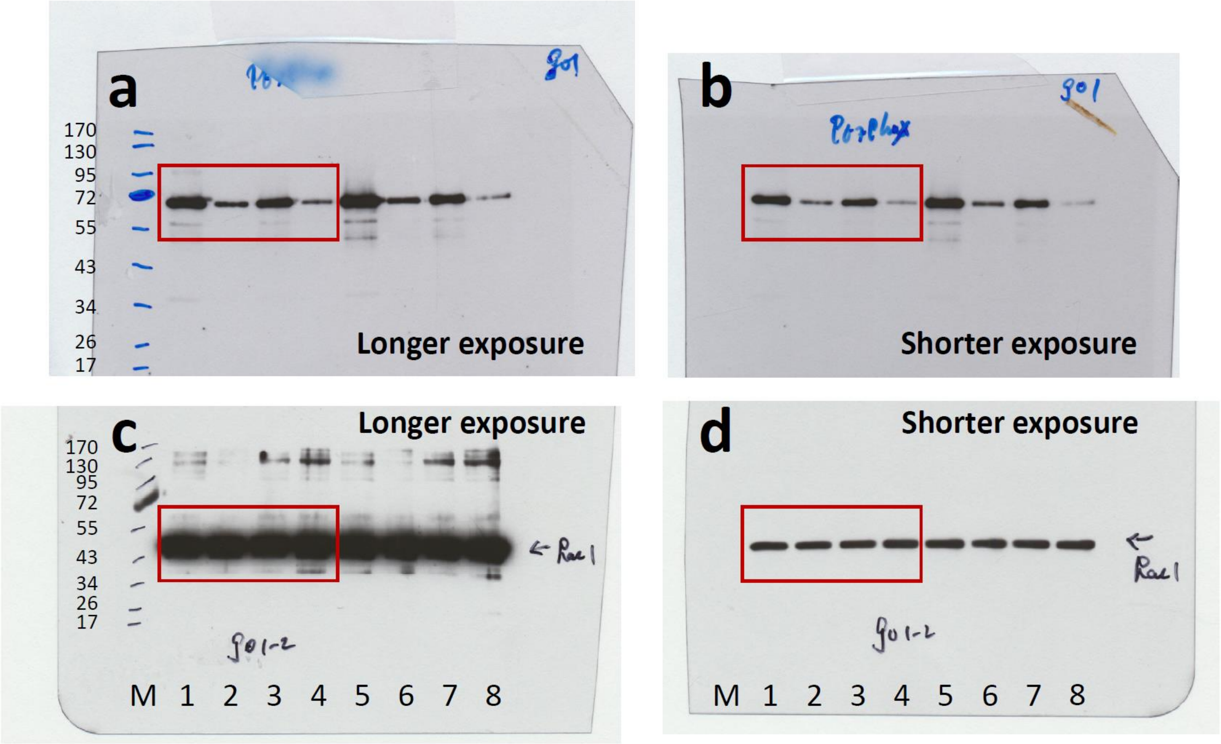

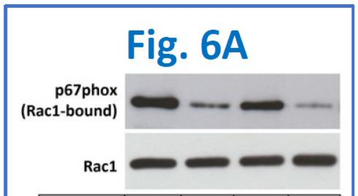

p67phox	+	+	+	+
Rac1	+	+	+	+
SIRT1	-	+	-	+

	1	2	3	4	5	6	7	8
p67phox	+	+	+	+	+	+	+	+
Rac1	+	+	+	+	+	+	+	+
SIRT1	-	+	-	+	-	+	-	+

Fig. S6. ***In vitro*** **deacetylation of Rac1 reduces its interaction with p67phox.** The Recombinant Rac1 protein (GST-tagged) was incubated with p67phox, with or without SIRT1, in a cell-free system. Then, Rac1 pull-down was performed with glutathione-agarose beads. The Rac1-pulldown samples were subjected to SDS/PAGE and transferred to nitrocellulose membranes.

(a) and **(b)**: The membrane was immunoblotted with an antibody against p67phox.

(c) and **(d)**: The membrane was blotted with an antibody against Rac1.

The red boxes indicate regions of interest for the main figure (Fig. 6A).
